# Carbohydrate Content Classification Using Postprandial Heart Rate Responses from Non-Invasive Wearables

**DOI:** 10.3390/s24165331

**Published:** 2024-08-17

**Authors:** Lucy Chikwetu, Rabih Younes

**Affiliations:** Department of Electrical and Computer Engineering, Duke University, Durham, NC 27708, USA; lc307@duke.edu

**Keywords:** wearables, mHealth, machine learning for health, carbohydrate content classification, dietary monitoring, automated dietary monitoring (ADM) systems, precision nutrition, diabetes management, postprandial heart rate responses

## Abstract

The rising incidence of type 2 diabetes underscores the need for technological innovations aimed at enhancing diabetes management by aiding individuals in monitoring their dietary intake. This has resulted in the development of technologies capable of tracking the timing and content of an individual’s meals. However, the ability to use non-invasive wearables to estimate or classify the carbohydrate content of the food an individual has just consumed remains a relatively unexplored area. This study investigates carbohydrate content classification using postprandial heart rate responses from non-invasive wearables. We designed and developed timeStampr, an iOS application for collecting timestamps essential for data labeling and establishing ground truth. We then conducted a pilot study in controlled, yet naturalistic settings. Data were collected from 23 participants using an Empatica E4 device worn on the upper arm, while each participant consumed either low-carbohydrate or carbohydrate-rich foods. Due to sensor irregularities with dark skin tones and non-compliance with the study’s health criteria, we excluded data from three participants. Finally, we configured and trained a Light Gradient Boosting Machine (LGBM) model for carbohydrate content classification. Our classifiers demonstrated robust performance, with the carbohydrate content classification model consistently achieving at least 84% in accuracy, precision, recall, and AUCROC within a 60 s window. The results of this study demonstrate the potential of postprandial heart rate responses from non-invasive wearables in carbohydrate content classification.

## 1. Introduction

As the global incidence of type 2 diabetes continues to rise [1], the demand for innovative and accessible dietary monitoring approaches has never been more critical. Technologies such as continuous glucose monitors have made significant strides in diabetes management [2,3]. However, their widespread adoption is mainly restricted to those already diagnosed, largely due to their invasive nature and high costs. This reality emphasizes the urgent need for non-invasive, innovative tools that promote early dietary awareness and enable timely interventions. Such tools are vital for individuals at risk or in the early stages of pre-diabetes, offering a pathway to initiate early dietary changes. However, the development of these technologies has not kept pace with the urgency of the need, thereby creating a gap in preventive healthcare [3,4].

Conversely, a growing body of research has consistently underscored the impact of dietary composition on postprandial heart rate variations [5,6], suggesting a strong correlation between the carbohydrate content of meals and subsequent heart rate changes [5,7,8,9]. This correlation is particularly pronounced within the first hour after eating [5,7,8,9], laying a solid foundation for the development of algorithms capable of classifying meals based on their carbohydrate content. Furthermore, such insights could be instrumental for decision support systems in diabetes management, potentially improving patient outcomes through personalized dietary recommendations tailored to individual profiles. Additionally, these technologies offer the possibility of providing users with detailed insights into their dietary habits through real-time alerts about carbohydrate-rich meals. This approach not only addresses the growing demand for carbohydrate-aware dietary monitoring tools but also opens new avenues for personalized dietary recommendations and diabetes management.

This paper investigates the use of heart rate data from non-invasive wearables to develop algorithms that classify whether an individual is consuming carbohydrate-rich foods (high-carb). While the application of machine learning in food classification is an established approach [10,11,12,13,14], utilizing heart rate responses to classify foods an individual has just consumed is an innovative area of research. Early approaches have primarily relied on visual [11,14] and acoustic [15,16] signals for food classification. This study is among the first to develop algorithms for classifying the carbohydrate content in meals based on postprandial heart rate responses, an idea suggested but not thoroughly investigated in Prioleau et al.’s 2017 review [17] of unobtrusive wearable systems for automated diet monitoring. While the use of physiological responses poses unique challenges, such as differentiating signals related to food intake from other physiological or environmental influences, it also facilitates an unparalleled degree of integration into everyday life, eliminating the requirement for active user involvement.

## 2. Materials and Methods

### 2.1. Application Development

**Figure 1:**
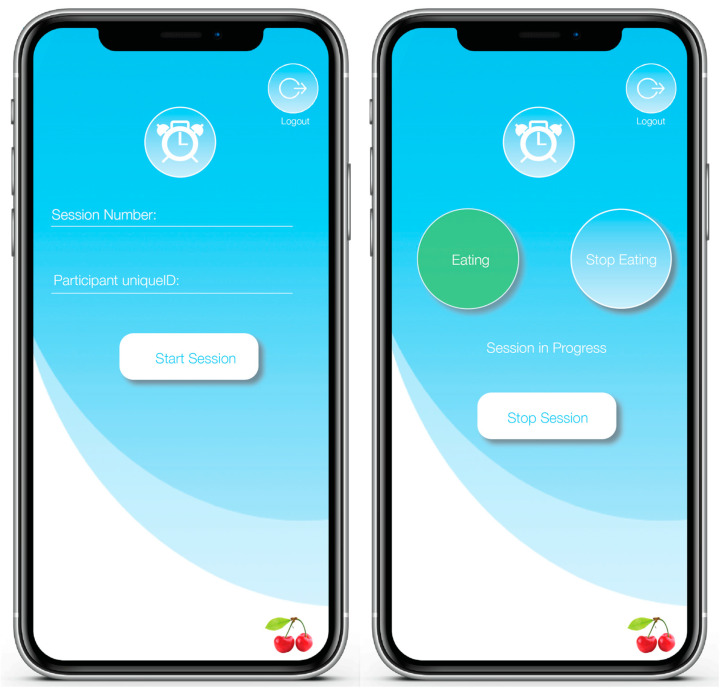
The timeStampr mobile application for collecting timestamps during data collection.

To optimize the performance of our models, we applied supervised machine learning techniques, which necessitated annotating our data with the start and end times of each eating class. We defined two eating classes: high-carbohydrate and low-carbohydrate. The high-carbohydrate (high-carb) class comprised eating-episode data when participants consumed carbohydrate-rich foods, while the low-carbohydrate (low-carb) class included eating-episode data when participants consumed low-carbohydrate foods. An eating episode was defined as a period where an individual is actively engaged in food or beverage consumption, characterized by actions such as lifting food to the mouth, chewing, and taking brief pauses not exceeding 15 s. To streamline data labeling, we developed timeStampr, an iOS application created using the Swift programming language within Apple’s integrated development environment—Xcode. The timeStampr application records the session number and a unique identifier for each participant. At the press of a button, it also captures timestamps to mark the start or end of an eating episode. At the end of each session, timeStampr generates a comma-separated file (.csv) of the captured timestamps. This file is subsequently saved in iCloud. Instead of relying on manual methods, such as checking a clock and recording the start and stop times of a participant’s eating episodes, timeStampr automates this process, thereby minimizing inaccuracies. Screenshots from the timeStampr application are displayed in Figure 1.

### 2.2. Study Details

We obtained approval from Duke University’s Institutional Review Board (Protocol #: 2023-0496) before commencing our study. Enrollment of study participants occurred on a rolling basis from September to October 2023. Our recruitment strategies included group emails, word-of-mouth referrals, and poster distribution. To be eligible to participate in the study, individuals needed to be healthy adults aged at least 18 years, residing in the United States, proficient in written and spoken English, capable of eating independently without assistance, and able to travel to Duke University for data acquisition. While our recruitment efforts primarily targeted the Duke community, we welcomed participation from individuals outside Duke who expressed interest in our study.

**Figure 2:**
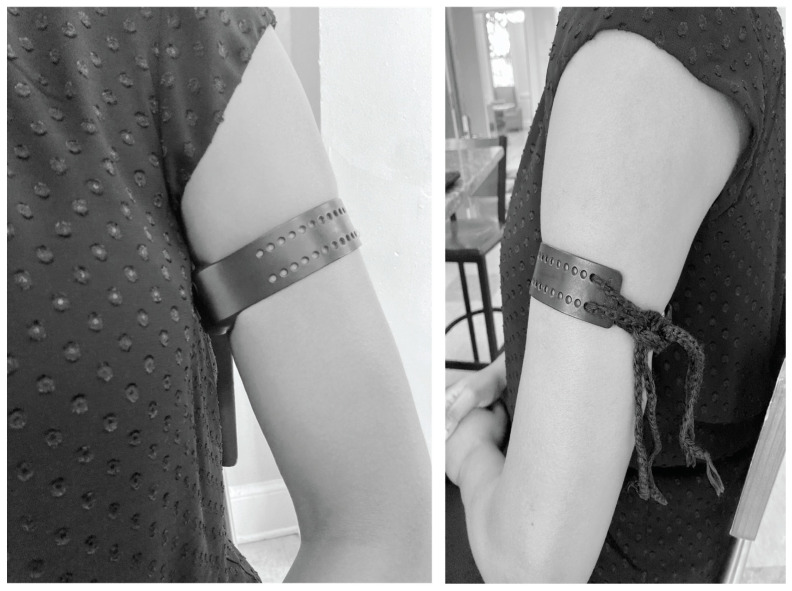
Empatica E4 positioning.

We collected data using an Empatica E4 device. It has a sampling rate of 1Hz for heart rate data. Although the Empatica E4 was designed for wrist-wear, participants wore the device on the upper arm of their non-dominant hand to minimize motion artifacts. We positioned the device facing towards the armpit and secured it with two strings for stability. Figure 2 illustrates the positioning. Although we collected data from 23 participants, we analyzed data from 20 participants for this study. We excluded data from two black males with very dark skin tones due to difficulties encountered by the photoplethysmography-based Empatica E4 device in collecting heart rate data. Additionally, we excluded data from another participant who disclosed a pre-existing heart condition, as our study targeted healthy individuals.

For the 20 remaining participants, 1 chose not to disclose their gender identity, 7 identified as male, and 12 identified as female. One participant was in the 30–39 age range, while nineteen fell in the 18–29 age range. Regarding ethnicity, one participant preferred not to specify their race/ethnicity, one identified as multiracial, three as white, five as black, and ten as Asian.

### 2.3. Data Collection

Individuals were required to review and electronically sign a consent form to participate in the study. After providing consent, participants completed an onboarding survey designed to provide the research team with demographic information, existing food allergies, and dietary restrictions. The study protocol consisted of two sessions per participant, during which data collection took place while participants were seated and consuming food. Details regarding the food consumed during these sessions can be found in Table 1. Taking into account our participants’ dietary restrictions and food allergies, we selected pineapple and banana for the high-carbohydrate session due to their widely acknowledged high carbohydrate content (≈27 g in a banana and ≈119 g in a whole pineapple). For the low-carbohydrate session, participants prepared bowls of arugula salad, each containing less than 5 g of carbohydrates per serving. Despite data collection occurring in a laboratory setting, efforts were made to create a naturalistic environment. Upon arrival at the laboratory, participants were assisted in wearing an Empatica E4 device on the upper arm of their non-dominant hand. Subsequently, we used Bluetooth to pair the device with an iPhone, which had the E4 real-time application for monitoring heart rate fluctuations in real-time. Once the Empatica E4 device was in place, participants were allowed to rest until the E4 real-time application indicated stability in their heart rate signal, typically achieved within a span of 10 min. After the heart rate signal stabilized, participants were provided with food, and training data were collected as they consumed food and took breaks. The start and stop times of eating were recorded using the timeStampr application.

We initially intended to standardize the durations of eating episodes; however, practical constraints led us to adopt a more flexible approach. Participants’ eating episodes varied in duration due to individual eating speeds and time constraints. Each session included at least two eating episodes, ranging from 1 to 10 min, with an average duration of 3 min, and interspersed with an average of 4.5 min devoted to non-eating activities. During non-eating intervals, participants engaged in non-eating activities of their choice, aligning with our goal of creating a naturalistic setting to enhance the study’s generalizability. Common activities among participants included texting, making phone calls, watching content on platforms such as YouTube, studying, writing, relaxing, and drinking water.

### 2.4. Feature Engineering

We utilized python’s tsfresh [18] package along with built-in feature selection techniques such as univariate feature selection and recursive feature elimination for feature engineering. For our final model, we selected 42 time and frequency domain features, including slope, mean, median, and maximum. Table 2 provides details of the features used in this work. In addition to automated feature engineering using python libraries and packages, we employed domain-specific knowledge to finalize the set of features. Features identified as relevant in prior projects or those that were theoretically viable, such as positive or negative slope values, were prioritized. Before implementing any feature engineering, we applied the sliding window technique with either a 25%, 33%, or 50% overlap. After experimenting with these window sizes, we settled on a window size of 60 s with a 25% overlap.

### 2.5. Classification

**Figure 3:**
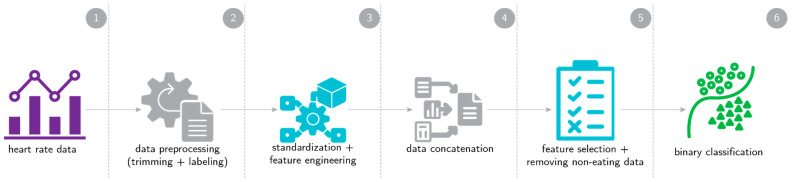
Carbohydrate content classification workflow: First, heart rate data are collected using an Empatica E4 device. The data are then trimmed to remove extraneous information, such as periods of rest before heart rate stabilization. Following this, the data undergo standardization and feature engineering, and the data from all participants are concatenated into a single dataset. Non-eating-episode data are removed, and feature selection is performed before running the binary classifier models.

To address intra-individual and inter-individual variations in heart rate, we standardized the session data of each participant independently before building any models. Timestamps generated by the timeStampr application were used to establish the ground truth. However, we excluded non-eating data and classified the remaining data as either high-carb or low-carb, depending on what participants were consuming during the data collection session. Figure 3 illustrates the classification workflow. Initially, we explored supervised machine learning techniques such as xgboost, random forest, and support vector machines for binary classification (high-carb versus low-carb). Subsequently, we investigated voting classifiers before returning to conventional machine learning techniques. Our final model utilized a Light Gradient Boosting Machine (LGBM) classifier with early stopping. To validate the model, we employed the leave-one-person-out (LOPO) cross-validation method, resulting in 20 cross-validation folds corresponding to our dataset of 20 participants.

### 2.6. Evaluation

The mode of the expected output served as the ground truth. We compared the output of each time window with its ground truth to assess our models. True positives (TPs), false positives (FPs), true negatives (TNs), and false negatives (FNs) were defined as follows:

TP: ground truth is high-carb and classifier predicts high-carb;

FP: ground truth is not high-carb and classifier predicts high-carb;

TN: ground truth is not high-carb and classifier predicts not high-carb;

FN: ground truth is high-carb and classifier predicts not high-carb.

We focused on five metrics: accuracy, precision, recall, F1 score, and ROC-AUC.

$$\mathrm{Accuracy}=\frac{TP+TN}{TP+TN+FP+FN}$$

$$\mathrm{Precision}=\frac{TP}{TP+FP}$$

$$\mathrm{Recall}=\frac{TP}{TP+FN}$$

$$F1\;\mathrm{Score}=\frac{2\times Precision\times Recall}{Precision+Recall}$$

## 3. Results

**Figure 4:**
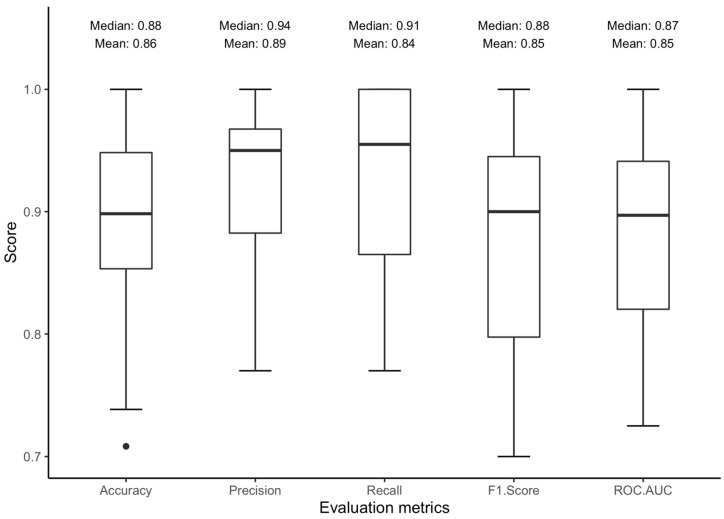
Boxplots illustrating the distribution of evaluation metrics obtained through leave-one-person-out cross-validation.

**Figure 5:**
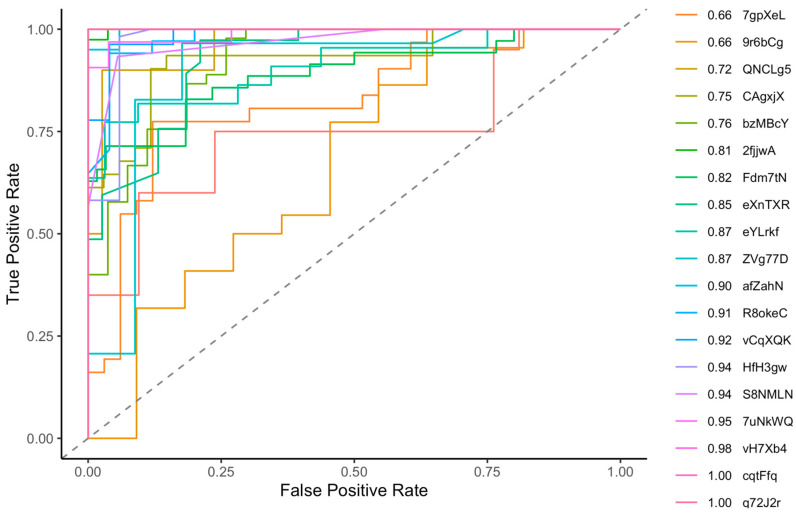
ROC curves from leave-one-person-out cross-validation.

In Figure 4, we display the distribution of key metrics (accuracy, precision, recall, F1 score, and ROC-AUC) achieved through leave-one-person-out (LOPO) cross-validation across the 20 folds of our final model. Each fold recorded an ROC-AUC greater than 70%, with the mean and median of all folds exceeding 84%. However, accuracy had two outliers, both pegged at 66%. Figure 5 shows superimposed ROC curves derived from the LOPO cross-validation. Each fold registered an ROC-AUC greater than 65%, and the mean and median values across the 20 folds were 85% and 87%, respectively. While each ROC-AUC score exceeded 50%, these results underscore significant inter-individual variations. The model exhibited superior performance when tested with data from certain individuals, while its performance notably decreased with data from others.

## 4. Comparison to Previous Work

While numerous studies have explored machine learning for food classification [10,11,12,13,14], leveraging an individual’s heart rate responses for this purpose represents a novel and innovative research direction. Traditional approaches have primarily relied on visual [11,14] and acoustic [15,16] data. To our knowledge, this study marks the first attempt to develop carbohydrate content classification algorithms based on individuals’ postprandial heart rate responses. Prioleau et al.’s 2017 review [17] of unobtrusive wearables for automated diet monitoring recommended monitoring blood pressure and heart rate as a promising research avenue, and our study actualizes this recommendation.

By leveraging physiological data, particularly heart rate, our approach presents the possibility of a less intrusive and more passive method for food classification. While utilizing physiological responses poses unique challenges, particularly in distinguishing signals related to food ingestion from other bodily or environmental factors, it also provides an unparalleled level of integration into daily life without requiring active user involvement. Due to the innovative nature of our approach, making a direct comparison with existing methods is not clear-cut.

## 5. Discussion

Our findings highlight the potential of heart rate monitoring in carbohydrate content classification. Achieving a minimum of 84% across key metrics—accuracy, precision, recall, F1 score, and ROC-AUC—based on data from 20 participants is noteworthy. These initial findings serve as a proof of concept for the application of heart rate monitoring in carbohydrate content classification. Implementing these findings into a practical application might pose fewer challenges than developing eating detection systems. This simplification arises from the fact that the carbohydrate content classifier is activated only during confirmed eating instances, eliminating the need to distinguish between heart rate fluctuations induced by food intake and those resulting from unrelated activities, such as emotional responses to music or physical exertion. It should be noted, however, that confounding activities such as stress and physical activity may still introduce complexities into these systems, especially when eating occurs while walking. While the eating detection framework used alongside the carbohydrate content classifier may not necessarily rely on heart rate, it is important to note that the carbohydrate content classifier itself does.

To advance this research, it is crucial to recruit a larger and more diverse participant pool. By including individuals with a wide spectrum of dietary patterns, health profiles, and demographic backgrounds, we can create a dataset that better captures the intricacies of physiological responses to carbohydrate intake. This inclusive approach does more than reveal diverse postprandial physiological responses; it significantly improves the generalizability of the carbohydrate content classifier, enabling its application across a broader population.

Furthermore, it is important to consider the interaction between food texture and heart rate. For example, crunchy foods such as raw vegetables require significant chewing effort despite having a low carbohydrate content. Therefore, examining how heart rate responds to chewing effort as opposed to the body’s metabolic response to carbohydrates is essential. Distinguishing between these different physiological responses will be critical for enhancing the accuracy and reliability of the classifier.

The pronounced variations observed in the ROC-AUC curves underscore the critical role personalized models play in carbohydrate content classification. Physiological responses to carbohydrate intake demonstrate significant individual variability, influenced by factors such as genetic predisposition, lifestyle choices, metabolic rate, and the postprandial glycemic response—the fluctuation in blood glucose levels after eating. This response serves as a key indicator of the body’s ability to process carbohydrates, which in turn impacts heart rate changes. By tailoring algorithms to account for each individual’s unique heart rate patterns, we can potentially achieve a significant improvement in the performance of our carbohydrate content classifiers.

## 6. Conclusions

In conclusion, the use of heart rate for carbohydrate content classification marks a new era in automated dietary monitoring. The initial success of our model establishes a solid foundation for a system that, with further refinement and customization, holds promise for significant progress in personalized nutrition guidance. Expanding the participant pool to include a wider range of demographics and dietary habits, while also considering different food textures, will enhance the model’s performance and generalizability.

Furthermore, advancements in carbohydrate content classifiers may pave the way for significant developments in nutrient and allergen sensing technologies that utilize non-invasive wearables. For example, food hypersensitivity can elicit significantly elevated heart rate responses and emotional arousal [19]. In such scenarios, wearables equipped with heart rate and electrodermal activity sensors, such as the Empatica E4, would be highly valuable. Integrating these technologies into eating-detection platforms could enhance the utility of these platforms and broaden the range of insights they provide.

## Figures and Tables

**Table 1:** Food consumed during each session.

	Consumed Foods
session I	$\frac{1}{2}$ pineapple and 1 banana
session II	make your own salad bowl (arugula, spring mix, red onion, cucumbers, green pepper, parmesan cheese, feta cheese, chicken, turkey, classic ranch dressing, Caesar dressing, and rich poppy seed dressing)

**Table 2:** Features engineered for time and frequency domains.

Features		
min	max	max−min
mean	standard deviation	median
interquartile range	values above mean	values below mean
average absolute difference	median absolute difference	skewness
kurtosis	number of peaks	energy
signal magnitude area	+slope values	−slope values
argmax	argmin	argmax−argmin

## Data Availability

De-identified datasets generated during this study are available upon request.
